# Foreign

**Published:** 1840-10-03

**Authors:** 


### MEDICAL EXAMINER.

DEVOTED TO MEDICINE, SURGERY, AND THE COLLATERAL SCIENCES.

No. 40.] PHILADELPHIA, SATURDAY, OCTOBER 3, 1840. [Vol. III.

### FOREIGN.

*On Subcutaneous Wounds.* By M. Jules Guerin.—In these papers, which form a summary of the very bold practice of the author in this novel branch of surgery, his chief endeavour appears to be to point out an explanation of the peculiar pathology of subcutaneous wounds; that is, of wounds of considerable extent made in all the tissues beneath the skin through a very narrow opening in it. The following abstract, however, contains what appears to us a more useful part of the memoir, and by showing to what an extent the practice may be safely carried will prove that it should be adopted in every possible case in which inconvenience is feared from exposure of the wounded tissues to the air.

"I took (says M. Jules Guerin) two dogs of moderate size, the one young, the other an adult. In the former I made a subcutaneous division of the mass of muscles in the posterior grooves of the vertebral column in three places; one in the superior scapular region comprising the trapezius, rhomboideus, serratus posticus, sacro-lumbalis, and longissimus dorsi, and the two layers of intertransversales; a second through all the muscles and aponeuroses in the middle of the back; and a third at the level of the third lumbar vertebra, implicating in like manner the whole width of the sacro-lumbalis and longissimus dorsi. After these, I cut transversely beneath the skin through the flesh of the posterior and upper part of each thigh quite down to the bone, so as to divide the glutæus maximus, biceps, semitendinosus, semimembranosus, vastus internus, adductor magnus, and the sciatic nerve and corresponding vessels. Each of these sections was made with a very narrow knife introduced through a small hole in the skin at some distance from the main incision; and to secure more effectually the exclusion of the air I closed the openings in the skin with sutures.

"In the adult dog I made a long wound under the skin, extending from the lower part of the neck to the sacrum, parallel to the line of spinous processes, so as to divide all the muscles down to the arches of the vertebræ. I then made a transverse section of all the tissues at the back of the left thigh from beneath the skin to the bone, as in the former case, and closed both the little openings in the skin with sutures.

"In the first dog immediately after the operation there were slight effusions of blood under the skin at the divisions of the vertebral muscles; and a considerable quantity of fluid poured out at those of the muscles and vessels of the thighs. In the second dog there was an effusion without any considerable swelling all along the hollow of the vertebræ corresponding to the wound, and an immense effusion in the thigh, so that the size of the buttock was doubled, and the skin was made round and tense.

"Both dogs passed the night quietly, and on the next day neither of them had the least appearance of fever. On the day after they appeared better, eating and drinking as usual, and beginning to move about freely. No trace of the operations on the muscles of the spine could be found in either of them, and in the thighs there was much less tumefaction and no pain on pressure. On the fourth day the oldest of the dogs seemed to have forgotten his mutilations; he was walking and running and jumping about all day, exactly as he used to do before the operations. Next day, however, his wounds were a little tender; but on the following he was again perfectly well, and every tissue that had been divided was united and perfectly restored to its former functions.

"In the younger dog the phenomena were still more interesting; none of the five wounds excited the least sign of inflammatory reaction: on the fourth day he began to raise himself up on his hind legs, (which had of course been completely paralyzed,) and on the eighth he could walk; he now presents scarcely the least trace of paralysis.

"Here, therefore, are examples of subcutaneous wounds implicating a great extent of tissues, dividing muscles, aponeuroses, vessels, and nerves, and followed by considerable effusions beneath the skin, and yet cured, nay even organized immediately almost, as if there had been but a simple lesion of the skin itself."

On the assurance afforded by the results of these experiments, M. Guerin has divided the human muscles to an almost equal extent in thirty persons affected with curvature of the spine. The operation was followed in all, he assures us, with considerable benefit; and on only one occasion did there result from it the least inconvenience; in that case there was an unimportant diffusion of air along the track of the wound.—*Brit. and For. Med. Rev., from Gazette Médicale.* Avril 1, 1840.

---

*Medical Experiments.* By Dr. J. C. G. Jörg.—About the year 1822 a society was formed at Leipsic for the purpose of trying the effects of medicines on the healthy: it consisted of Dr. Jörg, an eminent accoucheur of that town, and twenty-six other persons, three of whom were ladies. The results of some of their experiments were published by Dr. Jörg; and the account is preceded by observations on the advantages to be derived to medical science from experiments of this kind, and by a descriptive list of the members of the club. Thus, No. 4 was Mr. Frederic Conrad Steinbach, aged twenty-six, from Pegau, tall and stout, of robust constitution and sanguine temperament; he took his degree May 13, 1823. No. 21 was Mr. Charles Ottomar Otto, aged twenty-two, from Weissenfels, tall and lean, of lymphatic constitution, and sanguineo-choleric temperament. No. 22 is Dr. Jörg himself, who gives the following account of his own physical conformation:

"I am forty-five years old, stout, of a middle size, robust constitution, and sanguineo-choleric temperament. I suffered in the autumn of 1810 from a protracted and violent bilio-nervous fever; at the end of that year, and until 1812, I had several quotidians and tertians; and in 1813 I had a miliary typhus fever, (*Kriegstyphus.*) Ever since these illnesses I perspire copiously, and from slight causes; I am also inclined to diarrhœa, but I rarely labour under it, because I avoid all such meats and drinks as would be likely to bring it on. My bowels, however, are open two or three times a day. In order to keep in very good health, I require a bit of hard work once a week or fortnight—an object which I am generally fortunate enough to obtain in the practice of midwifery." (p. 25-6.)

The ladies are described in the following manner:—Ch., a lady aged forty-five, of short stature and delicate constitution, but healthy, and of a sanguine temperament. L., a young lady of eighteen, tall and slender, of a delicate and arterial constitution, a sanguine temperament, and quite healthy. Th., a girl aged twelve, of a middle size, and stout for her age, of an arterial florid constitution, and sanguine temperament; she likewise was perfectly healthy. Both the last, adds Dr. Jörg, live with the utmost simplicity: they eat meat and vegetables, but take neither wine nor coffee, and but rarely tea; they usually drink water, milk, or a weak pale ale; and, though they work and study, do not neglect exercise in the open air.

"When we tried experiments with any remedy, we followed no other dietetic rules than those by which we had been guided previously. We lived regularly in every respect, just as a healthy man must do, who wishes to remain healthy. The students of Leipsic, though their board is quite sufficient for the purpose of nourishment, do not find the dishes too numerous, nor prepared with too much art, so that they are not likely to be hurt in this way. Many of them drink coffee, but, partly by the roasting, and partly by the boiling, this has lost its narcotic power, and hence does not exhibit those poisonous effects which the exaggerated representations of many physicians would make us believe that it produces upon the healthy. Most of the students in Leipsic take wine only on rare occasions; and though a few of them drink beer, it is, for the most part, a very weak beverage, not capable of destroying the effects of the remedies experimented on.

"But these things are of less consequence than is commonly supposed, provided the ordinary course of life is adhered to; as for myself, I lived as usual during my experiments. At 5 or 6 in the morning I took two cups of coffee with milk; at 7 or 8, generally speaking, a little white bread and butter; at 1 I dined on soup and meat, with or without vegetables, and at 8 or 9 I supped in the same style. Both at dinner and supper I have long been in the habit of taking half a bottle (or a pound) of white Würzburg, or red Assmannshaüser wine; and I continued to do so during the experiments. Had I altered my usual mode of living, had I given up wine and coffee, I should have been out of *tune*, and perhaps made myself half ill. The result justified my mode of proceeding; for most of the medicines had the same effect on me as on the other members of the experimental society. Nay, in many cases, it was quite clear that medicines had less effect on those individuals who drank milk, white beer, or plain water, than on me who use other beverages. If any of us slept ill at night, we intermitted the medicines until due rest had restored our body to the proper degree of sensibility." (p. 21-23.)

The medical substances, of which the effects are reported and commented upon, are seventeen in number; namely, nitre, cherry-laurel water, bitter almond water, Vauquelin's prussic acid, Von Ittner's prussic acid, valerian, serpentaria, the flowers of arnica, the root of arnica, camphor, castor, musk, St. Ignatius's bean, assafœtida, opium, digitalis, and tincture of iodine.

Unless the contrary is expressly asserted, it is to be understeod that the dose mentioned was taken once only in the day. Although, in order to comprise this abstract within reasonable bounds, we must in general content ourselves with giving the results, and pass over the details of the experiments by which they were arrived at, we will make a single exception to our rule, and give the commencement of the numerous trials on which Dr. Jörg has founded his theory of the operation of nitre.

"On the 27th February, 1822, at 9 A. M., Engler swallowed a grain of nitre, rubbed down with five grains of white sugar, but without any effect.

"On the 2d of March, at the same hour, he took three grains of nitre, and six of sugar, and a few hours afterwards his urine was more copious, darker, and redder. After the lapse of several hours a sediment formed, which, when the urine was shaken, rose in flocculi. On March 4th, at the same hour, Engler took four grains of nitre, with the same quantity of sugar; this dose was followed by the same increased secretion of urine, and by a still more frequent desire to go to stool, without more frequent evacuations of the rectum. The same effects were produced by five grains taken on the 5th of March, and six grains on the 6th. On the 7th of March seven grains were taken at 9 in the morning, and again at 5 in the afternoon; on which the experimenter observed throughout the day that a pale and turbid urine was frequently discharged, and that there was repeatedly a sensation of pressure downwards to the anus, without more than the usual evacuations.

"On the 8th of March he omitted the nitre; the urine was reduced to its regular quantity, and the pressure towards the anus was no longer felt. Two doses, of eight grains each, being taken on the 9th of March, at 9 A. M., and 5 P. M., the same sensations were experienced as on the preceding day, though not in a greater degree than from the smaller doses. On the 11th of March he took two doses of ten grains each, at the same hours as before, and the urine becme still redder and more turbid, but was not secreted in greater quantity. Though there was repeatedly the sensation of bearing down towards the anus, the evacuation did not take place as usual in the morning, but towards evening. On the 12th of March two eleven-grain doses were taken at the same hours, and with the same results. On the 15th of March he took two fifteen-grain doses at the same hours, which were followed by an increased discharge of clear urine. The tenesmus and bearing down, however, were felt less frequently than after the earlier doses." (p. 28-29.)

The largest doses were taken by Assmann; on one occasion he took two drachms at once, and on another day two drachms at twice. These large quantities produced headach, violent thirst, oppression of the stomach, abdominal pain, and other distressing symptoms, so that Dr. Jörg dissuaded him from taking larger quantities. Dr. Alexander, of Edinburgh, however, whose experiments our author quotes at some length, tried the effects of nitre with much greater boldness, not to say rashness. He found that nitre is less active when it has been some time dissolved in water; when it was in this state he could take six, eight, or twelve drachms within twenty-four hours, without inconvenience, the only effect being an increased secretion of urine. But when he took an ounce dissolved immediately before swallowing, at the rate of a drachm every ninety minutes, in four ounces of water, the following were the effects. First, a refreshing coolness, then coldness and pain in the stomach, and at last sharp and lancinating pains, not only in the stomach, but through the whole body, which were so violent that for fifteen minutes he could not breathe without feeling the acutest pain at each inspiration. He afterwards divided an ounce and a half of nitre into eight equal parts, and took one every ninety minutes, immediately after its solution in water; but the pain in the epigastric region and over his whole body was so great, that he was obliged to desist from his experiments. Dr. Jörg then gives at some length a case of poisoning with nitre, related by Alexander; but we will avail ourselves of the brief abstract of the same case given by our great British toxicologist:—"A woman in the second month of pregnancy, immediately after taking a handful of nitre in solution, was attacked with pain in the stomach, swelling of the whole body, and general pains; she then miscarried, and afterwards had the usual symptoms of gastritis and dysentery, united with great giddiness, ringing in the ears, general tremours, and excessive chilliness. She seems to have had a narrow escape, as for three days the discharges by stool were profuse, and composed chiefly of blood and membranous flakes." (Christison on Poisons, p. 162-3.)

From the sum total of these experiments Dr. Jörg concludes, that *nitre is a stimulus to the kidneys, the intestinal canal, and the skin.* Its action upon the kidneys, which is most to be depended on, is shown by its increasing the quantity, and altering the quality, of the urine. It acts upon the alimentary canal from its commencement, and upon the auxiliary organs which open into it, increasing the secretion of saliva, causing dryness of the mouth and the œsophagus, thirst, morbid hunger, and pain in the stomach, (the pain being like that of inflammatory irritation,) a sensation of pressure or cutting in the small intestines, with rumbling, flatulence, and relaxation of the bowels; sometimes there is constipation, when the nitre has acted more especially on the small intestines, the kidneys, or the skin. Its power over the large intestine appears from its so often causing a desire to go to stool, even when this was not followed by actual evacuations. It more rarely affects the skin. When used in moderate doses it has no after-effects, and does not attack other organs. It was only when taken in very large doses that it excited giddiness or pain, or confusion of the head. Nitre, however, has one subordinate effect, which physicians have prized too highly, and have consequently prescribed it in diseases where it was totally out of place. When taken immediately after it has been dissolved in water, it cools the mouth, the œsophagus, and sometimes the stomach, for a few minutes. This primary effect, however, is soon followed by its secondary one, that of warming, namely; and the greater the former, the more marked is the latter. Thus the more the frequency of the pulse has been diminished by the original refrigeration caused by the medicine, the more is it increased by the subsequent reaction.

From this fact, and from its power of stimulating the skin, the kidneys, and the intestinal canal, Dr. Jörg deduces the conclusion that nitre is not an antiphlogistic remedy, but that it is to be used as a derivative in inflammation of the head, neck, and sometimes of the thoracic cavity. Moreover, as in moderate doses it does not act upon the nerves or the brain, it is well qualified to be a substitute for the mercury which is so frequently given too liberally in the phrenitis of children. In many cases, too, it must be an excellent emmenagogue. In the majority of cases the dose should be from three to five grains twice a day, though there are occasions when eight, ten, or more, should be given.

*Cherry-Laurel Water.*—The medicine used in Dr. Jörg's experiments was prepared in the following manner, in conformity with the prescription of the Saxon Pharmacopœia, (Dresden, 1820, p. 168.)

> Take of fresh cherry-laurel leaves cut into pieces lb. j.; alcohol ℥j.; common water lb. vj. After mixing, three pounds are to be distilled over.

The doses varied from three to fifty drops; though one experimenter, Heisterbergk by name, who appears throughout as the Mithridates of the club, took, on one occasion, 112 drops, with but little effect, save that of lowering his pulse about twelve beats in a minute.

Before recapitulating the effects of cherry-laurel water, Dr. Jörg observes that many drugs are tasted or smelt long after having been swallowed; while this substance, the moment it reaches the parietes of the stomach, seems decomposed, and therefore deprived both of its taste and odour. He remarks likewise that no drug throws more difficulty in the way of the experimenter than this one; for as it begins by attacking the head, it diminishes his capability of judging of his own sensations.

The cherry-laurel water was found to cause heaviness, with oppressive and stabbing pains in the brain, especially in its anterior part, in the region of the nerves of the eye, but particularly confusion of the head, with diminished sensibility of the whole body, slowness of the pulse, weariness and inclination to sleep, sleep itself, relaxation of the whole body, but especially of the thighs, disinclination to work; and, secondarily, irritation, itching, and tickling in the larynx, as in the commencement of inflammation of this part, frequent slight coughing, and increased secretion of a tough mucus in the trachea. It would appear, too, that the slowness of the pulse is more or less associated with the affections of the head.

This remedy acts as an excitant upon the brain; and, therefore, the retarding of the circulation and the diminution of the sensibility, for which physicians most frequently prescribe it, are purchased by an exaltation (though but a transient one) of nervous and cerebral life (*des Nervenund Gehirnlebens.*) Hence this medicine is ill adapted for patients suffering under inflammation or congestion of the brain, but will be advantageous in inflammation of the abdominal and genital organs, or in increased sensibility without inflammation. It must be injurious in pneumonia, as it has a tendency to inflame parts connected with the lungs, namely, the larynx and trachea, exciting cough and dryness of the mouth.

It will be particularly beneficial in diseases of the female genitals attended with increased sensibility, whether they bear an inflammatory character or not: on the other hand, it might do much harm in the first six days of scarlet fever, and in puerperal fever with a tendency of the materia lactea* (*Milchstoff*) to the head, as in either case it might favour the metastasis to the brain.

Dr. Jörg asks whether the cherry-laurel water, from the prussic acid which it contains, may not be additionally useful in the inflammations in which he has recommended it, by rendering the blood more venous, and, therefore, less plastic, and thus, more or less, preventing the adhesion of the inflamed parts by exudation. This effect would ward off great evils in inflammation of the intestines, peritoneum, urinary bladder, and uterus.

No one, he says, who is accurately acquainted with its medicinal properties, will believe that this remedy can have been of avail in obstructions of the abdomen, in what is called an atrabilious state, in hæmorrhoidal affections, in obstructions of the glands, in induration or carcinoma of the uterus. In these and similar diseases it can have done no real service, unless we reckon as such its depressing the sensibility of the nerves. Nor must it be used in the various spasms for which it is prescribed by physicians, without the greatest caution. Those spasms or convulsions which depend on irritation of the brain, or arise from repletion of its vessels, and the general or partial pressure which the central organ of the nervous system thereby suffers, cannot be quieted by this remedy.

As it acts upon the healthy in very different doses, the physician must select those for the sick with the greatest care; but they will probably range from three to fifteen drops, two, three, or four times in the twenty-four hours, though in some cases they must be gradually increased. We must recollect, too, that it soon loses its strength by standing, and by frequent opening of the phials in which it is contained.

*Bitter Almond Water.*—The medicine used in the experiments was prepared according to the Saxon Pharmacopœia, by mixing one pound of bitter almonds with an ounce of alcohol, and six pounds of water, and distilling over three pounds.

---

* By this phrase Dr. Jorg must mean the tendency to the head of that portion of the blood which, in a healthy woman, should secrete the milk.

It appeared, from the majority of the experiments, that although this remedy tasted and smelled more strongly of bitter almonds than the cherry-laurel water, it is weaker. Its effects, too, are of the same kind, and it may, therefore, be very well dispensed with.

*Vauquelin's Prussic Acid.*—The doses taken by the society varied from half a drop to three drops in twenty-four hours. They also destroyed four young magpies, two young cats, two young rabbits, a guinea-pig, and two frogs, with this drug. Four drops were sufficient to kill a half-grown male cat in three or four minutes.

It appeared, from examination after death, that, when prussic acid has been taken, the blood puts on a venous character, and is accumulated in the veins and the right half of the heart. Dr. Jörg also found this effect gradually take place in a frog poisoned with five drops, and placed under the microscope.

He concludes, from his experiments on men and animals, that prussic acid acts not only with the utmost rapidity, but with the utmost violence as a stimulus to the brain and nervous system, but more rapidly and violently on the the cerebral than on the ganglionic nerves. This excitement is sooner or later followed by a diminution of nervous and cerebral life, or by death itself. When death does not soon take place, an inflammatory irritation of the trachea, and especially of the larynx, is produced. It also paralyses the lungs, thus causing those dreadful sensations which arise from the non-oxygenation of the blood. Hence the advantage of taking the patient into the open air.

Dr. Jörg would wish to see this remedy expunged from the materia medica, on account of its dangerous strength; and we agree with him. The cherry-laurel water is far preferable. If Vauquelin's prussic acid is to be used, the dose should not exceed half a drop, or a drop, every four, six, or eight hours.

He remarks that, in all cases where the primary and secondary effects of a remedy are almost diametrically opposite, much depends on the magnitude of the doses and of the intervals between them: thus when some abdominal organ is violently inflamed, if it is desired to make the pulse slower, and the blood more venous, much larger doses of prussic acid will be required than if it is desired in a nervous disease to change the tone of the nerves.

*Ittner's Prussic Acid.*—The doses taken of this medicine varied from half a drop to three drops. Dr. Jörg himself took none; for Vauquelin's acid had acted so strongly upon his sensorium and common sensation, that he foresaw that Ittner's would have deprived him of his tact, both in the literal and figurative sense of the word. He found, however, from his experiments on men and animals, that this solution of prussic acid is rather stronger than the former one, and, therefore, still less deserves a place in the pharmacopœia. If ever prescribed, the dose should be from the fourth of a drop to a drop, every four, six, or eight hours.

*The root of Valerian.*—The society first tried an infusion; the proportions being generally from ʒij.—℥j. to a pound of water, and the time of maceration a quarter of an hour. The dose was usually a quarter, but sometimes one half, of this infusion. Sometimes it was taken in a more concentrated form, as for instance, ʒij. of valerian infused in ℥iss. of water.

When taken in the form of powder, the doses varied from half a drachm to two drachms and a half.

The results of the experiments show that valerian is a stimulus to the brain and the organs of digestion: when used in infusion it acts more on the head, when in powder, more on the abdomen and its organs. Dr. Jörg blames the manuals of materia medica for recommending valerian in hypochondriacal and hysterical cases. It is to be used, he says, in cases of debility, but not where there is any congestion of blood in the brain or the abdomen. The range of doses is to be from half a drachm to two drachms.

*The root of Serpentaria.*—An infusion was first tried; the quantity infused varying from a scruple to two drachms. The society afterwards took the powder in doses varying from gr. xv. to ʒiss.

Dr. Jörg found that serpentaria is a stimulus to the intestinal canal and its auxiliary organs. and that it favours congestion in the abdominal viscera; but with this peculiarity, that it does not promote mucous or glandular secretions, but rather the development of air in the intestines. Sometimes it acts as a stimulus to the brain, and causes congestion in that organ; sometimes it accelerates the circulation; and occasionally it stimulates the urinary, and, probably, also the genital organs. When infused, it acts more on the head and less on the abdomen, and *vice versâ* when in powder. When taken in small doses its effects last from 8 to 12 hours; when in large, from 18 to 20. Hence it should not be given oftener than twice in twenty-four hours; and sometimes once is enough. The dose should be from a scruple to a drachm, whether infused or in powder.

Dr. Jörg thinks it probable that serpentaria from its power of checking the mucous and fluid secretions of the intestinal canal, will be found particularly useful in chronic diarrhœa without any trace of inflammation, and in diarrhœa arising from colliquation. After observing that from its power of extricating air in the intestines, it would probably be hurtful when meteorismus is already present, he concludes with a very proper saving clause: "but this last point must be learned from experience at the sick-bed." (p. 181.)

*The Flowers of Arnica.*—This remedy was taken in infusion. The dose of the flowers varying from 1¼ to 45 grains, the smallest doses being taken by Mrs. Ch. and Miss L.; the largest by Heisterbergk. It was afterwards tried in the following manner. A drachm of the flowers was infused in six ounes of water, and a table-spoonful of the filtered infusion taken every two or three hours; this being the quantity and the intervals, says Dr. Jörg, in which most writers recommend this remedy, when it is to be taken in moderate doses.

He concludes from the experiments, that arnica flowers irritate and inflame the alimentary canal throughout its whole extent; the œsophagus, however, the stomach, and the small intestines, more than the large ones; that they act more upon the muscular fibres of the intestinal canal than on its vessels, and therefore promote contraction in the several divisions of the intestines, far more than secretion or absorption. They must also act upon the urinary system, and increase the secretion of urine either in quality or quantity; nor do the brain, the circulation, the skin, the lungs, or the trachea, escape the influence of this stimulus.

The action of arnica flowers lasts much longer than is commonly supposed, as it extends to from 24 to 36 hours.

Almost any book of materia medica will serve to show that arnica flowers are prescribed in doses too large, and too closely following each other.

If the patient is very irritable, a spoonful of fluid impregnated with the virtues of one or two grains of the flowers will suffice; to less sensitive persons we may give half an ounce of fluid, in which from three to five grains have been infused.

Dr. Jörg having found that arnica flowers are an external irritant, recommends them or their infusion as a mild rubefacient, and the infusion as an application to foul, malignant ulcers, which threaten sphacelus or induration.

*The root of Arnica.*—This drug was first tried in the form of a tincture prepared with one part of the root to six of alcohol. The doses were pushed as high as 84 drops, but the effects were very slight. It is rather to be considered a spirituous bitter, than as possessing the peculiar powers of arnica. The club then took the infusion of the root; the quantity infused varying from gr. iiss. to ʒj.

Dr. Jörg states the following differences between the infusion of the root and the infusion of the flowers of arnica. 1. The former being less acrid is a lesser stimulus to the cavity of the mouth, to the œsophagus, stomach, and small intestines. 2. Its slower and milder action upon the intestinal canal affects the muscles rather than the internal mucous membrane, and therefore promotes contraction, rather than any other function. 3. In persons whose digestive organs are not very irritable, it apparently acts more upon the brain than the infusion of the flowers.

Dr. Jörg concludes by remarking that arnica has long been celebrated for its power of discussing indurated parts, and promoting the absorption of fluid effused in the brain, but that this power was not shown among the experimenters, as there was nothing to be discussed or absorbed. Still, he says, the experiments clearly prove that arnica has this power; for, whatever increases the activity of the alimentary canal, exalts the function of the lymphatics, and causes derivation from the brain. Hence arnica is similar in its effects to calomel, with the difference that the former calls forth an inflammatory diathesis, while the latter rather promotes a scorbutic relaxation.

(*To be continued.*)

---

*Remarks on Continental Education—Florentine School—Bufalini.*—My Dear ———: As I have already said, the German manner of clinical education is in use in the Tuscan schools; but even in Florence the whole system of their transmontine allies is not fully developed in regard to it. The real distinction on this point, betwixt the Universities of Austria and Tuscany, is the total neglect on the part of the latter of those specialities in the study of medicine which forms so fine a featue of a continental education in this branch of science; we may, therefore, say, without hesitation, or fear of outraging truth, that the once free and genial Italian has basely copied from his conqueror all the ills of a forced education, and refused through indifference the excellencies it offers, which national genius had attached to it at an earlier period. Special pursuit in the cultivation of our art is no mere dream of the imaginative mind, nor yet the short conceit of empiricism; for we find our reasonable countrymen accept it as a practical good in the daily routine of the physician, whilst the philosophic German acknowledges its value as a truth, in adopting it *a priori* in his schedule of courses of study. We must not heed so much the new tendency springing up amongst some of our colleagues in France in favour of an opposite doctrine, since these are, for the most part, men of opinion, and strive at a false unity in things, and too great simplicity of system. It is an easy task to act the roll of physician here; it is only necessary to make a fair diagnosis—to maintain the trifling value of remedies, and effect a belief of danger attached to those more potent—to praise the curative powers of unaided nature, and in fatal cases to admire how many morbid processes have conspired to steal away the life of the patient. The zealot is a thousandfold better than the sceptic in all matters; so also in medicine.

The Florentine medical and surgical clinique present respectively about thirty beds; eighteen in the females' wards, and twelve in that of the men. This is about the utmost number of patients one can attend to, according to the manner of the Germans. The cases are in general well chosen, for the Florentine teachers are strongly doctrinary, but not eclectic like the Germans; and hence we find them much more zealous physicians. To defend dogmas, and maintain personal opinions, arouses men to work with promptitude and energy. Bufalini, the clinical professor in the medical department, is an ingenious physician, and author of the theory of localization; a set of views on pathology, founded much on truth; but, like all other theorists, he has carried his generalisations beyond their scope. His therapeutic agents are of course greatly modified by opinion, and we find in him neither the striking boldness of Tommasini, and the followers of Rasori, nor the timid trifling of the German school, nor the scepticism of the French. He seems a sort of middle man, who yet leans more to the confidence of the new Italian. I was sorry, however, to find him treat his patients on grounds merely logical; and the unavailing nature of his mode of cure sometimes made me wish it were a holy mandate that physicians must adhere strictly to the dictates of experience. There were other cases, however, in the treatment of which he borrowed largely from such authority; one of them was a woman of about forty-nine years, attacked by canine madness; she had been bitten by a rabid dog, eight months previous to her admission into the hospital. After the usual premonitory symptoms, the febrile stage began, accompanied with the horror of water and delirium. The pulse, at first increased in quickness, then gradually decreased, becoming lower and less frequent until the fifth day, when the unfortunate sufferer sunk in death; the other febrile phenomena progressed accordingly. Here our English authors were the chief guides in the treatment, and calomel, with opium and acetate of lead, were used without avail, till, at length, the injection of warm water into the veins proved of value; it quickly relieved the horror of water, almost curbed the delirium, and calmed the sufferings of the poor creature, Canine madness was now no longer a fearful disease; the physician could command its pains and its horrors; but the patient terminated her mortal career at the usual term of the malady, yielding peacefully her life, as all those do who die from progressive failure of the heart's action. Blood was drawn from the arm twice, each time previous to the injection, in order to guard against excessive plethora which might thence result, and to afford the means of a chemical analysis, as well as to fulfil some hopes of its proper utility in the cure. There is one peculiarity in the blood in this disease: it is found to contain much free prussic acid, independent altogether of any secondary change, or the trifling quantity naturally present in this fluid under the ordinary circumstances. It is sometimes very considerable; at other times less so; but it remains for future observers to prove if its presence be constant; therefore it would be too hasty to detect some relation between its development in the circulating mass, and the gradual failure of circulating centre and disordered functions of the mind. It seems, however, only to appear after the febrile stage is ushered in; and this is quickly followed by collapse. The mental disorder, in this case, also bears the nearest resemblance to the delirium of typhus, which is, for the most part, devoid of organic lesion, or even of morbid change of any kind in the vessels and solids of the brain; and the opinion that this depends on some fine alteration in the composition and qualities of the blood, which may act like narcotic medicines, (for there are animal compounds that do so,) becomes daily more probable. The autopsy was made most carefully, and with unwonted neatness; the preparation of the nerves, leading from the cicatrized wound on the back of the right hand to the brachial and cervical flexus, was nicely done; yet no perceptible alteration was found in any part. The brain, spinal marrow, lungs and air-passages, stomach and alimentary canal, liver, spleen, pancreas, kindeys, urinary passages and bladder, and all the parts appertaining to the abdomen, and elsewhere, were more than usually sound and healthful looking. Dr. Bardsley tells us he always found little red ulcers in the mucous membrane of the stomach; now, in this instance, I examined it most minutely, and found not even the minutest trace of any thing bearing the slightest shade of likeness thereto, nor yet blood-shot vessels; if any thing was remarkable, it might be a layer of very transparent, glairy mucus—an usual attendant on nausea, and the operation of such medicines as exert a powerful sedative action on the circulation. These ulcers, seen by him, were probably accidental, and, may be, such as we often find in the stomachs of English patients after all febrile complaints, and possibly owing to peculiarity of constitution and diet, and medical treatment.

Bufalini and his assistant use the stethoscope fairly and well; but he does not fail sometimes to make too hasty conclusions in diagnosis on its authority. Whilst I was in Italy he resolved a case of disordered action of the heart in an adult man to be hypertrophy to an unusual extent, and, after treating his patient a long time on this supposition with a half drachm of tartar emetic daily, the poor wretch died from a composition of maladies. The autopsy proved, in opposition to his opinion, that no hypertrophy or morbid alteration of structure of the heart was discoverable by any one of the five senses. Any reasonable man in our country would have admitted the commission of a fault of diagnosis in such circumstances; but not so the sanguine Italian professor, as he directly imagined he had cured an extensive hypertrophy by the large doses of the active antimonial! Never supposing for a moment the possibility of an error in the means of forming his first opinion, he came to the school the following day, and delivered a brilliant lecture on the case, when he declared to his enchanted class, amid enthusiastic greeting, the glorious deed accomplished; the enlargement of this noble organ corrected perfectly by the heroic administration of tartar emetic; and henceforth the news was sent from Florence to all Italy. He was determined not to doubt in the least his particular use of auscultation, even in one instance; and the old and accomplished Testa was here proved to have written his celebrated work on the maladies of the heart in vain; he, although he lived before the time of the stethoscope, would not have spoken so wildly, nor made such a great error in judgment. He would have simply seen one of those cases of nervous and sympathetic anormal action of this organ, which are most especially manageable by means directed against the primary disease, and to the improvement of the general health, and specifically by the use of iron, either in form of sulphate, as our Abercrombie recommends, or still better, in the form of hydrocyanuret, or ferrocyanurate of this metal, as generally employed by the Italians.

We have two means of judging the physician; by his success and errors; and as Bufalini is destined to be famed in foreign parts, I have shown him to you under both colours, to guard you against that fatal charm which forms a sacred halo around a foreign authority. "I will write it down—'tis meet it should be known"—we have the best physicians at home; but alas! the man of opinions will still remain the many-people's man.

There is an excellent madhouse in Florence, and a distinct division for skin diseases, in the Military Hospital, for all classes of patients; but these are not all available to the school in a clinical view, since there is no clinique on diseases of the mind or of the skin, nor any on those of children, or of the eye, nor yet an obstetric clinique. This last hospital offers the means, among the soldiery, for clinical instruction on the cure of syphilis, and this is likewise left unheeded: thus custom governs men, and governments rule amiss. The German style is therefore followed without its completeness. On the whole, however, the Tuscan physicians, together with the Genoese, obtain the longest practical education in their art of all continental nations, beyond France.

Very sincerely yours,

*London Medical Gazette, Sept.* 4.

---

*Clinical Lectures on Erysipelas delivered at the Hospital of La Pitié, Paris.* By M. VELPEAU.

### LECTURE 1.

*Various diseases confounded with erysipelas—Erysipelas properly so called—Angioleucitis—Phlebitis—Phlegmonous erysipelas—Causes.*

Erysipelas, although very frequently met with both in medical and in surgical wards, is one of those affections which is the least understood. If, indeed, we submit to a severe analysis the various descriptions of erysipelas which authors have given, it soon becomes evident that they have confounded under the same name various diseases; and this accounts for the discrepancy that exists between them with respect to the treatment of the malady. The ancients appear to have confounded, under the term erysipelas, even a greater number of inflammatory affections than modern writers, as is proved by the multiplicity of names under which it is designated. Thus, we find it described by them as the sacred fire, the red fever, the rose, St. Anthony's fire, &c. These terms, however, were evidently applied not only to erysipelas but also to other eruptive diseases. At a later period, and indeed in our own times, erysipelas has been confounded with erythema, with various bullous and dartrous affections, with angioleucitis or inflammation of the lymphatic vessels, and with external phlebitis. As long as this confusion lasts it is impossible to form a correct opinion of the nature of the disease; it is equally impossible to decide what plan of treatment ought to be adopted, as some of these affections are easily cured, whereas with the others it is quite the reverse. J. Frank, for instance, informs us that erysipelas is extremely frequent in Lithuania, and that it readily yields to judicious treatment. But on examining the account he gives of the disease, we recognise, instead of erysipelas, over which no plan of treatment appears to exercise much influence, a simple erythema, such as we often meet with in young persons who are arriving at the age of puberty, and in all old people, badly fed, or of a deteriorated constitution. If we really wish to understand the nature and treatment of erysipelas it must be separated from the other diseases with which it has hitherto been confounded. this we will endeavour to do.

Setting aside erythematous, bullous, and dartous affections, the diagnosis of which is too simple to require comment, I shall proceed to the examination of the anatomical and physiological characters of erysipelas itself, and of those affections which are most commonly mistaken for it, and also most frequently combined with it, viz., diffusive phlegmon, or phlegmonous erysipelas, angioleucitis and phlebitis.

### ERYSIPELAS, PROPERLY SO CALLED.

This, the simple or real form of erysipelas, may be recognised in many of the descriptions which authors have given of the disease. The characters which it presents are seen to the best advantage on the limbs. When it attacks the genital organs, the perinæum, &c. these characters are much less distinct, owing to the subcutaneous cellular tissue in these regions being generally more or less infiltrated with fluid, when the skin, which is extremely thin, is inflamed. The following are the anatomical symptoms of the malady: the skin suddenly assumes a red colour, the redness presenting itself in patches slightly raised above the level of the healthy parts. The patches do not, however, acuminate, but appear as if they were applied on the skin, between the cutis vera and the epidermis. The tumefaction and the redness do not decrease gradually, as in other inflammations of the skin, but terminate abruptly. The line of demarcation between the healthy part and that which is affected is, therefore, exceedingly well marked. The colour of the inflamed skin is generally a yellowish red, but it is subject to variation according to the constitution of the individual, or the state he is in when attacked. With persons of a lymphatic temperament, who have been weakened by loss of blood, the skin is sometimes of a milky yellow. In cases of this nature, you will find that although the skin presents this white appearance it offers all the other physical characters of erysipelas : the tumefaction might not inaptly be compared to a liquid poured over the healthy surface. This slight but general tumefaction of the skin, accompanied by the irregular festooned margin, is not met with in any other disease. The subcutaneous cellular tissue does not appear swollen, except in some few regions, as I have already stated. If the skin is attentively examined you will often find minute vesicles, some as small as a pin's head, some larger. These vesicles frequently increase in size so as to form phlyctenæ, and when this occurs the erysipelas has been called bullous. The skin is the seat of burning heat, and becomes acutely sensible, contact with foreign bodies giving rise to more pain than in any other cutaneous inflammation.

Before the local phenomenon make their appearance there are nearly always general symptoms, the intensity of which varies. These premonitory symptoms are similar to those which are observed in eruptive fevers, such as scarlatina or variola. Indeed, it is exceedingly difficult, in this stage of the disease, to say what it will eventually be ; the constitution of the patient, the state in which he is at the time, and other circumstances, must guide us in forming our opinion. The patient is first seized with chills or rigors, to which succeed great heat, burning thirst, and extreme restlessness. These symptoms are often accompanied by nausea, vomiting, sometimes by violent abdominal pains. They may be present from one to seven days, and generally persist, and sometimes, indeed, become more violent when the erysipelas has appeared. In variola, &c. on the contrary, three days after manifestation of the general symptoms, the eruption breaks out, and as soon as it has taken place they disappear, or at least are sensibly mitigated.—Ataxic or adynamic symptoms may be present, as in all other eruptive fevers.

When once developed the progress of erysipelas is peculiar. It may be termed a creeping or ambulatory disease, the red patches never remaining in the same spot, but gradually spreading to the adjoining parts. In some cases, however, it is called fixed, when, for instance, it attacks the head; but the entire surface of the head can never be affected at once.

The creeping nature of erysipelas must always be kept in mind, as it exercises the greatest influence over the duration of the disease. Each patch of inflammation lasts but four or five days ; consequently, were the eruption to take place at once on the entire surface which is to be affected, the malady would terminate in the course of a week or ten days. As, however, the patches appear successively and may, gradually progressing, invade the entire surface of the body, the duration of erysipelas is very uncertain. It may thus perpetuate itself during a month or more, and when this is the case, even this, the simple form of erysipelas, becomes a very dangerous malady.

Erysipelas, as characterized by the anatomical and physiological symptoms which I have enumerated, may be early recognised and separated from other diseases. We will now briefly examine the characteristic symptoms of Angioleucitis.

### ANGIOLEUCITIS.

Angioleucitis, or inflammation of the lymphatic vessels, differs in nearly every respect from erysipelas, with which it has lately been confounded by many writers. It may either be spontaneous or secondary : that is, it may appear under the influence of a peculiar state of the economy, or it may be the result of local injury.

The forms under which it presents itself are extremely diversified. When the consequence of a prick, a wound, &c., the lymphatic vessels of the wounded part are often inflamed before the ganglions to which they direct their course, and give rise to regular, red, ribbon-like streaks. These vascular streaks follow a tortuous course, extending in the direction of the ganglions either a few inches, or the entire length of the limb, and do not offer any tumefaction either to the eye or to the touch. Thus, when the hand, arm, or forearm, are the seat of the lesion, the superficial lymphatics are generally inflamed primitively, and it is only subsequently that the ganglions of the axilla become tumefied and painful. In other instances, on the contrary, the ganglions appear to be first affected. When, however, this is the case, a close examination will generally show that some vascular streaks are to be found in the vicinity of the lesion. The patches are irregular ; sometimes there are several, sometimes there is only one. Their colour is a livid red, not a yellowish red, as in erysipelas. The skin is smooth and even, does not present any vesicles, and seems rather more elevated in the centre than at the circumference, so as to form a slightly acuminated surface. These patches also terminate insensibly, so that it would be difficult to point out the precise spot where the skin ceases to be inflamed. In erysipelas, on the contrary, as we have already seen, the tumefaction is universal; indeed, if one part is more elevated than another, it is rather the circumference than the centre. In erysipelas the tumefaction ceases abruptly, and appears superficial, as if it were existing between the cutis vera and the epidermis, and not underneath the skin, as in angioleucitis. In this latter disease there are generally several patches of inflammation, which although sometimes united by inflamed streaks are often separated from one another by healthy tissues, whilst in erysipelas the inflammation may extend irregularly in various directions, but nearly always remains connected with the principal seat of inflammation.

If we now examine the physiological symptoms, we shall not find the same difference existing between the two diseases. The premonitory symptoms are nearly the same; indeed, before the eruption has appeared, it would be difficult to determine whether erysipelas or angioleucitis is about to declare itself. Thus angioleucitis is often preceded by thirst, nausea, fever; in a word, by all those symptoms which are observed in eruptive fevers. When, however, we find one or more ganglions tumefied and painful, there is reason to believe that angioleucitis and not erysipelas will ensue, unless there be erysipelatous patches already existing. It has lately been asserted by some authors, and among others by J. Frank, that tumefaction of the ganglions of the neck, accompanied by fever, is a certain symptom of impending erysipelas; but the assertion is by no means correct, as the cervical ganglions often become tumefied without erysipelas supervening; indeed, any injury done to the head will give rise to their tumefaction. The origin of this opinion may easily be accounted for. Erysipelas of the face and scalp is very frequent, and if the inflammation remains confined to the scalp, the nature of the affection is often not recognised at first. The ganglions, nevertheless, become inflamed, and when the erysipelas does become visible their tumefaction is considered to have preceded its invasion. It is, therefore, evidently difficult, in some parts of the body, to distinguish angioleucitis from erysipelas during the first period of the malady. Still, if attention be paid to the pathognomonic characters of these diseases, a correct diagnosis may be formed, even in such cases as those to which I allude. When cervical ganglions become tumefied, and the fever is but slight, it is most likely that angioleucitis will supervene. When, on the contrary, the general symptoms run high, the tumefactions of the ganglions generally indicate erysipelas.

The more we recede from the commencement of the disease, the more we find that these affections differ. The erysipelatous patches only last, as I have already stated, four, five, or eight days, and if the disease is of longer duration, it is that, under the influence of a general morbid state of the economy, the inflammation successively attacks parts previously healthy. Erysipelas may terminate by superficial suppuration, the serosity contained in the phlyctenæ becoming purulent; but never by suppuration or gangrene of the subcutaneous tissue, as in that case it is no longer simple but phlegmonous erysipelas. In angioleucitis each patch constitutes as it were a phlegmon, and may run through all the periods of phlegmonous inflammation, terminating by resolution, induration, or gangrene. Nor is the inflammation always confined to the superficial lymphatic vessels; sometimes it penetrates the deep-seated tissues, invading the lymphatics which are situated beneath the aponeurosis. The anostomosis which exist between the two layers of vessels at once explain this extension of the inflammation. The superficial lymphatics, and the cellular tissue which surrounds them, are first inflamed, and an abscess is formed: the inflammation is then propagated to the deeper-seated vessels, a new phlegmon taking place, and this may continue until the limb is penetrated in various directions by a chaplet of abscesses, if I may be allowed to use the term. Angioleucitis, when arrived at this stage, cannot be mistaken for erysipelas.

PHLEBITIS.

Phlebitis is a disease which has been confounded with erysipelas by J. L. Petit and others, although the difference between the two is even greater than that which we have found to exist between erysipelas and angioleucitis. Since, however, the spirit of analysis has become more prevalent, most practitioners have learned to distinguish them from one another. Yet even now the inflammation of the cellular tissue which surrounds the veins, or external phlebitis, is occasionally mistaken for erysipelas. I need scarcely say that it is only with the external form of inflammation that this affection could possibly be confounded; internal phlebitis, followed, as it rapidly is, by purulent absorption, and accompanied by the usual train of symptoms, is too well defined a disease to be mistaken for a cutaneous affection. External phlebitis generally occurs in the arms or legs, after bleeding. The premonitory symptoms are but slight in most instances, merely consisting in a little pain and uneasiness of the part. The cellular tissue which surrounds the inflamed vein, as also the adjoining integuments, becomes swollen and red, and patches are thus formed along the course of the vein. These patches appear deep-seated, and if they are examined with the finger it will be found that they rest on a kind of knotty cord, which is the inflamed vein. They communicate with one another, but differ, nevertheless, from the patches we meet with in angioleucitis. These latter are, it is true, united by red streaks, but they do not present the cord I have just mentioned, and are scarcely perceptible to the finger. In external phlebitis, the ganglions are not generally painful, whereas in angioleucitis this pain in the ganglions is one of the first symptoms that appear. Erysipelas is even more easily distinguished from this affection than from angioleucitis. Indeed, it is only because the characters of erysipelas itself were not well defined, that the two diseases have been confounded. Scarcely any of the symptoms, of that affection are to be traced in external phlebitis, neither the precursory symptoms, nor the uniform tumefaction of the skin, nor its irregular festooned border.

In external phlebitis, as in angioleucitis, the inflammation may terminate by resolution, but the resolution is a much longer process than in the latter complaint, the veins long continuing to form knotty cords.

PHLEGMONOUS ERYSIPELAS.

We have now to consider phlegmonous erysipelas, or diffused inflammation of the subcutaneous cellular tissue. a much better term than the one usually employed. Were it adopted, the word erysipelas might then be reserved for the simple inflammatory affection of the skin which we have just described. This is one of the most serious diseases that surgical practitioners have to treat. It has only been separated from other affections within the last thirty years. It was first described at length by Duncan, and then by Dupuytren, and has, since then, been much studied, especially by hospital surgeons. It is, however, evident, from the treatment apopted by various practitioners, and from the various results which follow that treatment, that it is not always the same malady that is held in view.

The seat of this disease is the subcutaeous cellular tissue. This tisssue is composed of two layers ; the first, the one in immediate contact with the skin, may be called the areolary layer. It is formed by cellular lamellæ, interwoven with one another, and adherent to the internal surface of the skin; it is a mixture of fibrous, cellular, adipose, vascular, and nervous lamellæ. Th second layer, that which is immediately applied to the aponeurosis, presents neither adipose nor cellular filaments, but offers a lamellated structure ; it is what Chaussier called "the lamellated element." When the inflammation is situated in the first layer, it extends with difficulty, owing to the structure of the tissue, and thus tends to produce a circumscribed phlegmon. When, on the contrary, the internal layer is inflamed, finding but little resistance to its progress, owing to the lamellated nature of the cellular tissue, and being confined between the denser cellular layers and the aponeurosis, the inflammation may extend rapidly, and invade the entire limb in the course of a few days. More or less effusion of liquid also takes place, and, as no organic fluid can long remain out of the vessels which naturally contain it, without changing its properties, it becomes troubled, and then purulent. The vessels of the areolary layer passing through the effused fluid, also soon become mortified.

Let us now examine the symptoms by which phlegmonous erysipelas may be recognised. When it supervenes as the consequence of a wound, there are seldom any premonitory symptoms. The edges of the wound suddenly assume a swollen and tumefied appearance, and the surrounding tissues become soft and boggy. The part affected presents a regular, deep-seated redness, similar to that of phlebitis, but differing from the venous redness of angioleucitis, or the yellowish redness of erysipelas. This redness disappears on pressure, and does not return as quickly as in the affections we have just examined. The tissues also retain the impression of the finger, which is never the case in angioleucitis or phlebitis.

The march of this disease is also peculiar. Like simple erysipelas, it progressively invades the adjoining tissue; but, unlike that affection, it does not abandon the regions which it has already attacked. In the course of five or six days the cellular element becomes mortified, and, if an incision is made, escapes under the form of white flakes, as in simple phlegmonous inflammation, which has terminated by suppuration.

If these characters are attended to, it will be nearly impossible to confound this affection either with simple erysipelas, or with the other two diseases which we have examined.

These four forms of inflammation are frequently united, and may, in that respect, be compared to the inflammatory affection of the eye. This circumstance must be always borne in mind, as their progress, their treatment, and their consequences, are essentially different. Simple erysipelas may be accompanied by phlegmonous erysipelas, by angioleucitis, or by phlebitis, and reciprocally. Thus a patient is seized, after the usual precursory symptoms, with simple erysipelas, and the affection, during a variable period, follows its usual course. The inflammation, however, is propagated more or less suddenly to the subcutanecus cellular tissue, and thus gives rise to diffusive phlegmon. This extension of the inflammation is more especially to be feared when the eyelids, the scrotum, the margin of the anis, &c. are effected, there being in these regions beneath the skin a very loose layer of cellular tissue. It is by no means difficult to ascertain when the complication has taken place. In simple erysipelas no swelling, to any extent, occurs ; and when the skin presents the tumefied acuminated appearance which I have already described, we may be certain that the cellular tissue is inflamed. Sometimes it is angioleucitis which appears as a complication of the original disease, the ganglions which communicate with the seat of the erysipelas becoming tumefied and painful. The patient is again seized with rigors, complains of thirst, want of sleep, &c. If the affection of the lymphatics is slight, this is all that occurs. If, on the contrary, it is more serious, the red streaks and patches of angioleucitis make their appearance, and the ganglions become indurated. It is, however, easy to see that the erysipelas and the angioleucitis are in reality two different affections, the characteristics of which may be distinguished, although they be combined. Lastly, either internal or external phlebitis may supervene. The symptoms of internal phlebitis, the small pulse, the prostration of strength, the drawn features, are too well known not to be at once recognised; and the red patches, resting on an inflamed cord, and accompanied by partial induration, will sufficiently characterise external phlebitis.

These three inflammatory diseases may also exist primitively, and the erysipelas itself appear as a complication. Thus, I have frequently seen patients suffering under angioleucitis suddenly seized with nausea, and an exacerbation of the febrile symptoms, followed by the appearance of the erysipelatous eruption. In internal phlebitis, the entire economy is so deeply modified, that the skin seldom becomes the seat of active inflammation. In external phlebitis, on the contrary, erysipelas often supervenes; and this is easily explained when we consider that there is a certain degree of inflammation already existing. In some instances the erysipelas appears to cure the affection of the veins.

You see, therefore, that these four diseases may exist simultaneously in the same individual, and that you may, nevertheless, still distinguish the characteristic symptoms of each. When this is the case, the angioleucitis will be the first to disappear, then the simple erysipelas, after which the phlebitis, and, lastly, the diffusive phlegmonous inflammation. We will now examine the causes and the nature of erysipelas.

*Causes.*—The causes of erysipelas are of two kinds; the determining, and the predisposing. The determining causes are every thing that can irritate the skin; the predisposing are more difficult to discover. Sometimes for two or three months we do not see in our wards a single case of erysipelas; whilst, at another period, the slightest prick, the slightest incision, the bite of a leech, the application of a blister, is sufficient to give rise to it. But, although we are inevitably led to admit a predisposing cause, we know not where to look for it. It is not to be found in the age, the sex, or the temperament of the patient: these predisposing causes being in action at all times, it is impossible to explain through them the appearance of erysipelas. Many authors have endeavoured to account for its manifestation by the action of external agents. Thus, some have attributed it to cold, because they saw it in winter; others, to heat, because they saw it in summer; whilst others account for it by a sudden change from heat to cold, or from cold to heat. Are we, however, authorized to look upon appreciable meteorological phenomena as the predisposing cause of a disease which is met with in every season? In the surgical wards of La Charité, for instance, erysipelas has raged this year during the winter; the year before, during the summer; and the year before that, during the autumn. As it is a disease which exists epidemically, some again have accounted for its appearance by the supposition of a deleterious principle contained in the atmosphere, which modifies the state of the constitution. Many writers maintain that erysipelas is a contagious malady. Lawrence, in England, Gibson, in America, have defended this opinion, which certainly has arguments in its favour. But these arguments are not sufficiently powerful to carry conviction with them, and we may yet consider the question as far from being satisfactorily settled.

If we examine the action of this predisposing cause, we find that it greatly modifies the disease. Erysipelas cannot certainly be looked upon as a simple inflammatory affection. It neither presents the characteristics nor follows the course of simple inflammation; nor is it possible to produce it artificially. Its course appears rather to resemble that of eruptive fevers; we may, therefore, conclude that, in erysipelas, the inflammation is not the real essence of the disease. The pale lactescent form of erysipelas, which we sometimes observe on extenuated patients, is an additional proof of the correctness of this view of the disease. We have now in our female ward a remarkable case of this form of erysipelas. The patient in question entered the hospital about a fortnight ago, labouring under a white swelling of the knee-joint, and disease of the uterus, and was so extenuated by continued hæmorrhage, &c., that one would have supposed it nearly impossible for an extensive cutaneous inflammation to appear. Yet, after being preceded by the usual symptoms, erysipelas made its appearance a few days ago. The skin, however, instead of presenting the usual yellowish red tint, is of a pale colour, scarcely different from that of the healthy integument, merely offering the slight general tumefaction with the irregular festooned border of erysipelas.

Erysipelas appears to be a general disease, the origin of which is in extensive alterations of the fluids; it is, consequently, to this general modification of the economy that the attention of the practitioner should be chiefly directed. To resume in a few words, erysipelas is but the shadow of a much more serious affection; and its gravity depends less on the extent of the inflammation than on the intensity of the general symptoms which have preceded or accompanied it.

Some authors deny that the cause of this affection is general, because it often appears without any precursory symptoms. We often, it is true, meet with it in surgical cases when nothing has forewarned us of its impending invasion; but this cannot be considered a serious objection.

The appearance of a disease due to a general cause is not necessarily preceded by general symptoms. If an alteration of the fluids exists, whatever may be the cause, we can easily understand that the economy may resist in the same manner as it would had a poison been taken, but in too small a quantity to disturb the regular exercise of the functions, and that, in its efforts to get rid of the deleterious agent, the skin may become the organ on which that agent is carried.

If we now examine the nature of the erysipelatous inflammation, we shall find that there are many opinions on that subject. Formerly it was said that the skin was the seat of the inflammation. At a later period, it was thought that it occupied the sub-epidermic tissue, and then again the capillaries of the skin were named. In our own times, the venous capillaries of the skin have been fixed upon as the seat of inflammation. This opinion has been defended in France by MM. Ribes and Cruveilhier; in England, by Dr. Copland. I have often minutely examined the skin in persons who had died of this disease, but never found any appearance which could warrant the supposition; nor do the arguments brought forward in favour of this opinion appear very conclusive. How, indeed, is it possible to prove that the inflammation exists in the venous capillaries, when it has never been possible to examine them. M. Ribes has found small veins underneath the skin filled with pus; but this was not owing to erysipelas, but to phlebitis followed by absorption of pus. M. Cruveilhier also speaks of having seen veins underneath the skin filled with pus; but he has never remarked them in the cutis vera. When we consider that erysipelas often covers a surface of several square feet, it scarcely can be allowed that the capillaries alone are inflamed. It is much more probable that all the tissues which enter into the structure of the skin, the nervous, the adipose, the cellular, the vascular, are simultaneously attacked. Indeed, no symptom seems to indicate that one of the various layers into which the skin has been divided is affected sooner than another; they are all the seat of inflammation, which often extends, as we have seen, to the subcutaneous cellular tissue. Indeed, if we look upon erysipelas as a malady occasioned by a deleterious principle which the economy contains being thrown on the cutaneous absorbing surface, we can scarcely understand even theoretically that one alone of the various elements which enter into the composition of the skin should be inflamed.

These views are of great importance with regard to the treatment of erysipelas. There is an immense difference between those practitioners who look upon the affection as local, and those who consider it to be attributable to an alteration of the fluids. In the eyes of the first, the treatment of the disease is already well known, and little or nothing remains to be done. With the latter, on the contrary, it is very different. Thinking that but little is known about the treatment of erysipelas, they feel convinced researches have yet to be made.

---

*On the Pain of the Back in Intermittent Fever.* By Dr. Grossheim. — This paper contains the results of observations made in fifty cases of intermittent fever, for the purpose of testing the opinions of Dr. Kremer, who had stated that a painful sensation is a constant and pathognomic sign of intermittent fever, on pressure over the first dorsal vertebra and the adjacent parts of the spine, and that there is a constant relation between the severity of this symptom and of the general disease. The following are the results which Dr. Grossheim has obtained, and which seem to confirm Dr. Kremer's statements, although they had been contradicted, as we formerly stated, by the experience of other observers: 1. Pain on pressure on some part of the spinal column is a constant symptom of intermittent fever, except in those cases in which the ligaments of the vertebræ have become by age or disease so rigid that they will not yield to pressure. 2. There is no definite locality to this pain; it may be situated in any part of the column, but is most frequent in the middle of the dorsal portion, especially in quotidian intermittents. 3. The extent of the pain also varies considerably; one or two vertebræ only may be tender, and the pain rarely occupies the space of more than five or six; it may also be situated at distant parts, with intervals in which none is excited by pressure. 4. The intensity of the pain is equally variable. Sometimes it was so severe that the slightest touch of one of the spinous processes produced severe suffering; but sometimes violent pressure was required to detect it. Among those vertebræ that excited pain when pressed, the middle one was commonly the most sensitive; and in those above and below it, the tenderness gradually decreased. 5. The pain was more severe during the paroxysms than in the intermissions. When the severity of the fever diminished, or the tendency to its returns grew less, the severity and the extent of the pain in the back also decreased; but the complete removal of the fever was not always accompanied by the entire loss of the pain, which often continued in a modified degree after the fever had ceased to return, and remained the longer the more severe it had previously been. 6. Complications of the intermittent fever did not appear to have any influence on the pain; it continued when the character of the fever was altered either for the better or for the worse, and it returned in cases of relapse.

From observing the constant existence of this symptom, the author was induced to try what would be the effect of remedies that would tend to correct the local excitement that seemed to exist. He relates very briefly five cases, in which eight or ten leeches were applied over the spine, in the situation where pressure gave the most pain. In four of these no other remedy was required; the pain ceased in a few days, and there was no recurrence of the febrile paroxysm.—*Brit. and For. Med. Rev.*, *from Medicinische Zeitung.*

*On the Presence of Iodine in Cod-liver Oil.* By L. GMELIN.—[The source of the therapeutic utility of this very nauseous medicine is in a great measure demonstrated by the following observations of Professor Gmelin.]

I had announced, says Professor Gmelin, that it was impossible to detect iodine in two kinds of cod-liver oil, of which one had a clear and the other a brown colour; and I had left it undecided whether the iodine found by other chemists had proceeded from the iodate of soda employed in their experiments, or whether certain samples of the oil did really contain iodine. The following researches will show that the genuine oil does clearly contain iodine, and that the examples of it which I first examined were spurious:

By treating sixty grammes of pure cod-liver oil, from Bergen in Norway, by Hausmann's method, I obtained by solution in alcohol a saline mass, which acted in the following manner: Its aqueous solution, mixed with starch and diluted sulphuric acid, gave a violet colour, which immediately disappeared on adding oil of vitriol, and was exchanged for a yellow hue. The same solution gave with starch and chlorhydric acid a violet colour, which soon disappeared on the addition of chlorate of potash. The experiment was repeated with 750 grammes of the same oil. The results were the same; only that in consequence of the greater quantity, the colouring of the starch was much more intense, and was not so promptly destroyed by the oil of vitriol or the chlorate of potash. Carburet of sulphur agitated with the aqueous solution of the saline mass with the addition of diluted chlorhydric or sulphuric acid, was coloured violet; and when a portion of the same saline mass was thrown into a mixture of peroxyde of manganese and moderately diluted sulphuric acid, which had been previously heated in a glass tube, the violet vapours of iodine were immediately seen rising and staining starch-paper blue.

These experiments leave no doubt of the presence of iodine in this cod-liver oil. On the authority of M. Tiedemann, a merchant at Bremen who has an intimate knowledge of the article, Prof. Gmelin says that there are in commerce four kinds of genuine cod-liver oil. The oil is melted by exposing the livers to the sun in casks which are placed upright, and divided into three parts by moveable boards placed one above the other. The clearest oil, and that which is most fit for medicinal purposes, is that which floats to the top; the next layers are coarser, and the lowest are quite brown. The refuse of the casks forms a deep coloured and thick oil, which is used in the manufacture of leather.

[In the Dublin Journal for July last, there is a valuable paper by Mr. Donovan, pointing out the great desideratum of rendering this oil palatable. He says this is effected by using a temperature in its expression not exceeding 192°.]—*Ibid., from Bulletin de Thérapeutique.*

*On the Danger of applying Leeches which have been previously used.* By M. PUCHE.—The transmission of virus by the fangs of leeches is an important question, and worthy of examination. M. Puche, physician to the hospital of Midi, has treated a patient in his wards, who affords a convincing proof of the danger of employing leeches which have been applied before. A messenger, æt. 24, was admitted into the hospital with urethritis of four months' standing, which had been recently complicated by acute inflammation of the epididymis. The epididymitis was the consequence of excessive labour, not of arrested discharge. Many applications of leeches were made to the hypogastric region for its removal. Five of the leeches applied were purchased at a low price by the nurse. Their bites inflamed, and took the aspect of Hunterian chancres. Now these syphilitic ulcers were too recent to have arisen from the same impure connexion that produced the gonorrhœa; but as it was possible that they were occasioned by the gonorrhœal matter coming into contact with the leech-bites, M. Puche, to satisfy himself, inoculated one part with the whitish discharge, and another part with the pus of the ulcers, on the 28th of February, 1840. On the 4th of March the inoculation of the urethral matter had produced nothing, while that of the chancres had given an ecthymous pustule, which had the regular development of syphilitic pustules, and terminated by an indurated and coppery cicatrix. It is argued that the ulcers proceeded from the leech-bites, (the leeches had certainly been employed on a syphilitic patient,) and that they had conveyed the infection from one patient to the other. The possibility of this transmission may be granted; but other experiments are necessary to prove it, and to determine whether the leeches are not destroyed by the virulent principle they imbibe; whether the poison is destroyed by them; and if so, at what period do they become innocuous.—*Ibid., from Bulletin Géneral de Thérapeutique.*

*On the Itch in Adults and in Children.* By Dr. KRAUSE, Physician to the Poor in Dantzic.—The chief object of this paper is to prove the inseparable connexion between itch and the acari, which many have supposed to be only occasionally present in that disease. The author gives examples to prove, what we believe has not been before noticed, that the disease may exist in those who wash themselves very often, or who have very tough skins, without any eruption; the itching and the power of communication may be present, but no visible sign of the disorder may exist, except the burrows of the insects. The following are his cases:

1. A journeyman tailor had three weeks previously slept with a comrade who had the itch. He came to me with a burrow in his left-index finger, from which I drew out an acarus, and I then examined him most carefully, but could discover no trace of any further eruption.

2. A servant girl, the skin on whose hand and forearm was scarcely inferior to leather in colour and thickness, complained of itching in it. By the side of the finger I discovered seven burrows, out of which I extracted three acari; but there was not a pimple or a vesicle to be found on the whole body.

3. A washerwoman, the mother of a numerous family, remarked that her two youngest children were constantly scratching themselves, and had an eruption on their hands. She showed me the children, and they had distinct itch, but she denied having it herself; she allowed, however, that she had sometimes felt an itching in the fingers and arms, but she had never remarked any vesicle. This statement was correct, for on the fingers, which had a kind of varnished appearance from the constant action of the hot water and the soap-ley, I could find no eruption, though there were several burrows, from which I pulled out four acari. On further questioning it appeared that the woman had long noticed these, but had thought nothing of them because there were no vesicles.

There are only two cases, the author says, in which one would not have a right to maintain that itch did not exist, when neither burrows nor acari can be found; namely, quite at the beginning of the disease, and when treatment has been employed for some days, and the skin is partially scaling off, so as to destroy the burrows and the acari, but not the brood, which may soon after generate a new eruption.

If one has the luck to draw an acarus by daylight, one may easily be sure of his existence by cracking him on the thumb-nail; he makes a noise just like, only weaker than, that which his near neighbour, the fair hexaped, makes in the same circumstances. But if one brings him close to the flame of a candle, he bursts with a distinct snap.—*Ibid., from Casper's Wochenschrift.*

---

*On the Employment of Lactate of Iron.* By MM. Gelis and Conte.—After stating some objections to the preparations of iron in common use, the authors give their reasons for supposing that the lactate of the protoxide of iron is superior to all other ferruginous preparations. These are that lactic acid is universally distributed throughout the body; and that all authors have endeavoured to administer iron in the form most easily soluble in the gastric juice. Berzelius, Tiedemann, and Gmelin, Dumas, Leuret, and Lassaigne, have shown that the gastric juice has sufficient lactic acid to account for its dissolving property. They find that the most useful preparations of iron are those most soluble in lactic acid and the reverse, and therefore consider it probable that iron after administration is converted into lactate of iron. Thus they were led to give the lactate direct.

M. Bouillaud, and other members of the academy, administered this preparation in twenty-one cases of anemia and chlorosis; but though they speak favourably of it, it did not appear to them to possess any decided advantage over other soluble preparations of iron.—*Ibid., from Bulletin de l' Académie Royale de Médecine.* Fevrier 29, 1840.

---

*On a New Species of Strangulation in the Tunica Vaginalis Testis, followed by Internal Strangulation.* By M. Laugier.—M. Laugier, surgeon to the hospital Beaujon, presented to the academy a pathological specimen, which is an extraordinary example of the above strangulation, caused by a circular fold of peritoneum, the edge of which surrounds the superior orifice of the inguinal canal, and the free border of which floats in the abdomen, forming a sort of prolongation of the inguinal canal. It was formed by a double fold of peritoneum detached from the anterior wall of the abdomen. It was easily passed into the tunica vaginalis (like the finger of a glove cut off) from ten to twelve lines. It was returned with equal facility. A portion of small intestine, about a foot in length, had pushed this production before it in passing into the sac of the tunica vaginalis, and became strangulated by the floating ring. The strangulation, therefore, was not at the neck of the sac, as usually occurs in congenital hernia; neither was it an encysted congenital hernia, for there was no secondary sac, but a ring at the free part of the floating fold. The reduction of the strangulated hernia produced the separation of the circular band which surrounded the intestine, and the external strangulation became internal, without there being any possibility of suspecting before death a similar course of strangulation in the tunica vaginalis and internal strangulation.

The dissection revealed the existence of a similar fold on the opposite side, and the patient had a congenital reducible hernia in the left groin. But as there was neither strangulation nor rupture, the free border of the fold was rounded, and not broken as on the right side. No similar disposition of parts has been hitherto recorded. If it had been possible to suspect it during life, it is not likely that an attempt at reduction would have been made, for the reduction itself was the cause of the strangulation in producing rupture of the fold. Gastrotomy was the only operation that could have been practised, but the rapid death of the patient prevented this.—*Ibid.*, *from ibid.* Juin 30, 1840.

---

*Congenital Dislocation of the Humerus reduced after Sixteen Years.* By M. Gaillard, Surgeon to the Hôtel Dieu, of Poictiers.—The following is an abstract of a report to the Royal Academy of Medicine, drawn up by MM. Cannet and Bouvier. They consider the case as very curious, and without a parallel in the history of surgery.

Mademoiselle L. B., a few days after birth, presented a deformity of the left superior extremity with much pain during its movements. The elbow was thrown from the body, the forearm semiflexed, and the hand in a state of pronation. During her infancy she could not bring the elbow near the trunk, nor raise her hand higher than the chin. The limb was often painful during her early years; but latterly the pain had only come on when the limb had been long fixed in one position. When M. Gaillard saw her in 1836 she was sixteen years of age. He found in the posterior part of the shoulder a projection formed by the head of the humerus, which was placed in the subspinous fossa of the scapula, above the middle part of the spine, which was curved by the pressure it had undergone. On moving the arm, the head of the bone was felt rolling under the fingers, and it appeared abraded from rubbing on an irregular surface. The hand could not be brought to a state of supination. The forearm could not be extended on account of the permanent contraction of the biceps. Elevation and rotation of the arm were impossible.

On the 5th of January, 1837, the patient was placed on a stool, a cushion was applied to the external border of the scapula, and maintained by two cords passing from its extremities and attached to two fixed rings. The arm directed horizontally inwards was then submitted to the action of a weight of sixteen pounds. The weight was suspended at the end of a cord which passed through a fixed pulley, and was attached to a bracelet fixed above the elbow. Time after time M. Gaillard increased the power of the traction, occasionally adding his efforts to the force exercised by the weights. The extension prolonged from twenty to twenty-five minutes was repeated on the 10th and 11th of January, each time bringing the head of the humerus nearer the glenoid cavity, and with no other inconvenience than a slight pain and swelling of the arm. On the 13th the head of the humerus was more moveable. Further traction was employed, the arm being always placed horizontally. The humerus yielded, glided over the scapula for the space of an inch and a half, and approached the glenoid cavity. M. Gaillard then seized the elbow, and by carrying it backwards and upwards he directed the head of the humerus downwards and forwards, then lowering the limb he felt the head pass under the acromial arch, and leap over a projection which appeared to belong to the articular cavity. The arm was now in contact with the trunk, it was sensibly lengthened, and all its movements could be executed with ease.

The dislocation, however, recurred again and again, and was as often reduced. The difficulty was to maintain the bone after reduction. This, however, was effected by careful bandaging. The patient suffered considerable pain at times, but was relieved by the application of leeches. From the first reduction to the entire cessation of pain a period of more than two years elapsed; but the cure was then complete, the patient using the limb equally as well as the other.—*Ibid.*, *from ibid.*

---

*Deafness after the Use of Quinine.*

To the Editor of the Lancet.

*Sir*,—I have observed the relation in your Journal of several cases of dumbness having been produced by the exhibition of quinine, and, as the cause has been doubted by your correspondent S. Y. G., in a former number of the Lancet, I take the liberty of relating a somewhat analogous case, although the effect produced was upon the sense of hearing, which I believe is by no means uncommon, and is clearly referrible to the same cause, the effect of a powerful tonic upon the nervous system.

I have lately had under my care a lady, the subject of gangrene of the lung, followed by circumscribed abscess, with profuse and fœtid expectoration. The treatment adopted after the abatement of the pneumonia in the first instance, was principally the exhibition of large doses of quinine, commencing with nine and getting up to thirty grains a day, which, with a little variation according to circumstances, was taken for several months successively. With the exception of a marked beneficial, though, unfortunately, but temporary influence over the disease, the only appreciable effect produced by these larger doses was a more or less complete state of deafness, which moderated when the dose was diminished, and disappeared entirely when the medicine was discontinued.

That quinine should have a powerful, though temporary effect upon the nervous system, is not to be wondered at, when we consider its specific effect in ague and some neuralgic disorders; and that its influence should be exerted in the peculiar manner stated over the nerves of speech or hearing, I do not consider either singular or alarming. My patient took nearly eight ounces in seven months.

Yours obediently, C. R. Bree.

